# Calorie Restriction Modulates Reproductive Development and Energy Balance in Pre-Pubertal Male Rats

**DOI:** 10.3390/nu11091993

**Published:** 2019-08-23

**Authors:** Guilherme Rizzoto, Deepa Sekhar, Jacob C. Thundathil, Prasanth K. Chelikani, John P. Kastelic

**Affiliations:** 1 Department of Production Animal Health, Faculty of Veterinary Medicine, University of Calgary 3330 Hospital Dr. NW, Calgary, AB T2N 4N1, Canada; 2 Gastrointestinal Research Group, Snyder Institute for Chronic Diseases, University of Calgary, Calgary, AB T2N 4N1, Canada

**Keywords:** caloric restriction, restricted feeding, realimentation, puberty, reproductive development

## Abstract

The objective was to determine effects of feed restriction and refeeding on reproductive development and energy balance in pre-pubertal male rats. Sprague Dawley rats (*n* = 32, 24 days old, ~65 g), were randomly allocated into four treatments (*n* = 8/treatment): (1) Control (CON, ad libitum feed; (2) Mild Restriction (MR, rats fed 75% of CON consumption); (3) Profound Restriction (PR, 50% of CON consumption); or (4) Refeeding (RF, 50% restriction for 14 days, and then ad libitum for 7 days). Feed restriction delayed reproductive development and decreased energy balance and tissue accretion, with degree of reproductive and metabolic dysfunctions related to restriction severity. In RF rats, refeeding largely restored testis weight, sperm production (per gram and total), plasma IGF-1, leptin and insulin concentrations and energy expenditure, although body composition did not completely recover. On Day 50, more CON and RF rats than PR rats were pubertal (5/6, 4/5 and 1/6, respectively; plasma testosterone >1 ng/mL) with the MR group (4/6) not different. Our hypothesis was supported: nutrient restriction of pre-pubertal rats delayed reproductive development, induced negative energy balance and decreased metabolic hormone concentrations (commensurate with restriction), whereas short-term refeeding after profound restriction largely restored reproductive end points and plasma hormone concentrations, but not body composition.

## 1. Introduction

Calorie restriction is a dietary regimen that reduces intake (20–40% reduction in calories compared to ad libitum consumption) without causing malnutrition [1], but reducing incidence of a variety of diseases, e.g., diabetes, cancer and autoimmune diseases [2]. Long-term calorie restriction or short-term starvation (fasting) reduces body weight and maximizes lifespan of rodents [3]. However, calorie restriction can have adverse effects on male reproduction, including reductions in sperm production, testis size, diameter of seminiferous tubules and sperm quality [4]. In that regard, adequate nutrition is fundamental for reproductive development and function in prepubertal males [5], with reductions in nutrient intake delaying reproductive development [6,7]. Furthermore, nutrient restrictions in females also reduce fertility [8], decreasing the probability of establishing and supporting a pregnancy when nutrient resources are deficient. However, subsequent access to ad libitum refeeding restores reproductive performance [9].

There is substantial evidence that pathways controlling energy balance and puberty are intertwined, as metabolic and neuronal sensors such as Kisspeptins, mTORr and GnRH-related neurons play an important integrative role [10,11]. Furthermore, overnutrition predisposes to obesity and male infertility, likely by dysregulating homeostatic mechanisms regulating energy balance and reproduction [12]. In a recent systematic review of 12 studies and ~1500 individuals, calorie intake and total energy expenditure were increased in non-obese pubertal adolescents, together with increases in fat and lean mass [13]. However, less is known regarding whether calorie-restriction induced impairments in male reproductive performance are associated with alterations in energy balance. Several metabolic hormones are important in growth and development [14]. For example, insulin-like growth factor-1 (IGF-1) and leptin reflect whole-body reserves, with reductions in circulating concentrations of these hormones often associated with delayed onset of puberty [5,7,15,16]. However, most feed-restriction studies have used adult animals, with very limited data on pre-pubertal animals, particularly males. Furthermore, we are not aware of any studies that rigorously characterized the changes in energy expenditure and substrate utilization with concurrent alterations in male reproductive parameters, during the pubertal transition period. Therefore, there is a need to study nutritional modulation in pre-pubertal males and determine its impacts on spermatogenesis, reproductive development and energy balance.

The objective was to determine effects of two levels of feed restriction and refeeding, with a focus on reproductive development and energy balance (i.e., energy intake, energy expenditure, substrate utilization and body composition), in pre-pubertal rats. We tested the hypothesis that nutrient restriction of pre-pubertal rats delays reproductive development, induces negative energy balance and suppresses plasma concentrations of metabolic hormones, whereas short-term profound feed restriction and refeeding restores reproductive end points, energy balance and hormones to levels similar to those in control rats with continuous ad libitum intake.

## 2. Materials and Methods

### 2.1. Rats, Housing and Experimental Design

Male Sprague Dawley rats (*n* = 32; Charles River, Montreal, QC, Canada) arrived with their mothers at Day 14 (birth = Day 0); they were still suckling on arrival and weaned on Day 21. On Day 24 (body weight, ~65 g), they were randomly allocated into four treatment groups (*n* = 8 per group): (1) Control (CON, ad libitum access to feed); (2) Mild Restriction (MR, rats fed 75% of diet consumed by CON); (3) Profound Restriction (PR, rats fed 50% of diet consumed by CON); and (4) Refeeding (RF, 50% restriction for 14 days and then switched to ad libitum for seven days). Starting at assignment to groups, rats were housed individually in metabolic cages (Comprehensive Lab Animal Monitoring System, CLAMS^®^; Columbus Instruments; Columbus, OH, USA) under controlled temperature (23–25 °C), humidity (22–24%) and lighting conditions (12 hours light–dark cycle; lights off at 22:30). Throughout the study, rats were fed a standard powdered rat chow (energy density 4.07 kcal/g; PicoLab^®^ Rodent Diet 20; LabDiet, St. Louis, MO, USA; [17]) and had ad libitum access to water. Animal use was reviewed and approved by the University of Calgary Health Sciences Animal Care Committee (#AC12–0033).

### 2.2. Metabolic Measurements

Feed intake and energy expenditure were recorded for ~22 hours daily using the CLAMS system from 22:30 to 08:30. Readouts measured by the system were volume of oxygen (VO2) consumed (mL/kg body weight/h), carbon dioxide (VCO2) produced (mL/kg body weight/h) and respiratory exchange ratio (RER) through indirect calorimetry (2 L/min flow and 48 min sample interval). Respiratory quotient (RQ), was obtained by dividing CO_2_ production by O_2_ consumption. Total energy expenditure was computed by the following formula: 3.815 × VO2 (L/h) + 1.232 × VCO2 (L/h; [15]). Once a week, body weight was recorded and body composition measured with quantitative magnetic resonance (Minispec LF-110^®^, Bruker Optics, Milton, ON, Canada).

### 2.3. Blood Collection and Hormone Analyses

On Days 36, 43, 50, 57 and 64, blood samples were collected from the saphenous vein into tubes containing EDTA (1.1 mg/mL blood), protease inhibitor cocktail (10 μL/mL blood, 1:100; Sigma-Aldrich, Oakville, ON, Canada) and dipeptidyl peptidase IV inhibitor (10 μL/mL blood, 50 μM; Millipore Corporation, Temecula, CA, USA; Singh et al., 2016). Blood glucose concentrations were immediately measured using a glucometer (Accu-Chek™ Glucose Meter; Roche Diagnostics, Laval, QC, Canada); the remainder of the blood sample was centrifuged (1000× *g* for 10 min) and plasma removed and stored at −80 °C pending additional analyses.

Plasma concentrations of leptin and insulin were quantified with a Rat Metabolic 5-Plex Features assay, whereas plasma IGF-1 concentrations were determined with a TM featured-mouse IGF-1 Array Single-plex assay. Assays for these three metabolic hormones were done by Eve Technologies (University of Calgary, Calgary, AB, Canada). Values above the upper limit (upper quartile + (3 × interquartile range)) or below the lower limit (minor quartile + (3 × interquartile range)) were considered outliers and removed from the dataset. For testosterone concentrations, a commercial assay was used (Testosterone ELISA kit, Enzo^®^, Lausen, Switzerland), with specificity of 100%. Assay sensitivity was 5.67 pg/mL, with inter- and intra-assay CVs of 11.3% and 10.0%, respectively.

In a previous study [18], 100% of Sprague-Dawley rats had testosterone >1 ng/mL, balano-preputial separation and presence of sperm at 48 days of age; therefore, in the present study, puberty was defined as testosterone >1 ng/mL and we determined the number of rats that had reached puberty on Days 50 and 64. Due to limited sample availability, testosterone concentrations were only measured in six rats per group for CON, MR and SR and five rats for the RF group on Days 50 and 64.

### 2.4. Testis Evaluations

Testes width was measured on Days 29, 35, 43, 50, 57 and 64. Rats were restrained on a table, with light pressure applied to the abdominal wall to ensure both testes were within the scrotum. With testes side by side, calipers were used to measure width of both testes (at the widest point), with values recorded to the nearest 1 mm. The same person performed all testes measurements.

After being on the study for 5 weeks, rats were euthanized in a CO_2_ chamber on Day 64. Testes were removed, carefully cleaned (to remove fat and connective tissue), epididymides excised and paired testis weight recorded. Thereafter, one testis of each pair was sectioned longitudinally, with one half fixed in Modified Davidson’s media (MDM [19]) and the remaining half plus the other testis stored at −80 °C for qPCR. Excised epididymides were placed in a 1.5 mL tube containing 1 mL of PBS at 37 °C, finely minced with scissors and placed on a warming plate at 37 °C for 15 min to allow sperm to swim out. An aliquot was collected to assess motility assay using a computerized sperm analysis (CASA) system (Spermvision^®^ Version 3.5.6.2, Minitube, Verona, WI, USA), with eight fields evaluated for each sample. The same sample was used to produce morphology slides stained with eosin/nigrosin [20] and unstained slides for acrosome integrity using FITC PSA [21]. For sperm morphology and acrosome integrity, 200 sperm per rat were assessed, without knowledge of treatment group. Number of sperm per gram of testis and daily sperm production (DSP) were determined as described [22,23]. A half-testis was weighed, ground in a Waring tissue blender, sonicated in 2 mL of Milli Q water and final volume brought to 15 mL by addition of Milli Q water. Elongated spermatids (resistant to homogenization) were counted and sperm production determined as described [23]. Furthermore, to obtain total sperm/testes, the number of sperm per gram of testis was multiplied by testis weight.

To determine seminiferous tubule diameter, testis samples stored in MDM were embedded in paraffin blocks, sectioned (5-µm thick), and sections mounted on slides, stained with hematoxylin and eosin and images digitized using a Leica RM 2500 microscope with camera. Using ImageJ^®^ software (National Institutes of Health, Bethesda, MD, USA), 100 tubules were measured for each rat, with cross-sectional averages used to compare groups [24].

### 2.5. RNA Isolation, cDNA Synthesis and Gene Expression Analyses by Real-time Polymerase Chain Reaction (RTPCR)

Total RNA (~100 mg) was extracted from 200 mg of testis using a commercial kit (RNeasy Mini Kit; Qiagen Inc., Toronto, ON, Canada) according to manufacturer’s instructions. After isolation, RNA was treated with Deoxyribonuclease I, Amplification Grade (Invitrogen, Burlington, ON, Canada) to eliminate DNA. Then, 1.0–1.5 μg of RNA was used for synthesis of complementary DNA (cDNA) on a Master cycler pro thermocycler™ (Eppendorf Canada Ltd., Mississauga, ON, Canada). Quantitative PCR was done as described [17,25] on a Mastercycler ep realplex thermocycler (Eppendorf Canada Ltd.). Each reaction (total volume, 25 μL) consisted of 12.5 μL of SYBR Green master mix (Applied Biosystems Inc., Foster City, CA, USA), 2 μL of cDNA, 0.2 μL of each primer (100 µM) and 10.1 μL of RNase/DNase-free water. Primers for target genes, namely IGF1 and IGF1R, were designed using the Primer-BLAST online tool (https://www.ncbi.nlm.nih.gov/tools/primer-blast/). Actin was used as a housekeeping gene. Sequences of primers and housekeeping gene are shown (Table 1). Relative differences in mRNA of these target genes were calculated as follows: ΔΔCT = ΔCT (sample) − ΔCT (calibrator) [26,27].

### 2.6. Statistical Analyses

**Figure 1:**
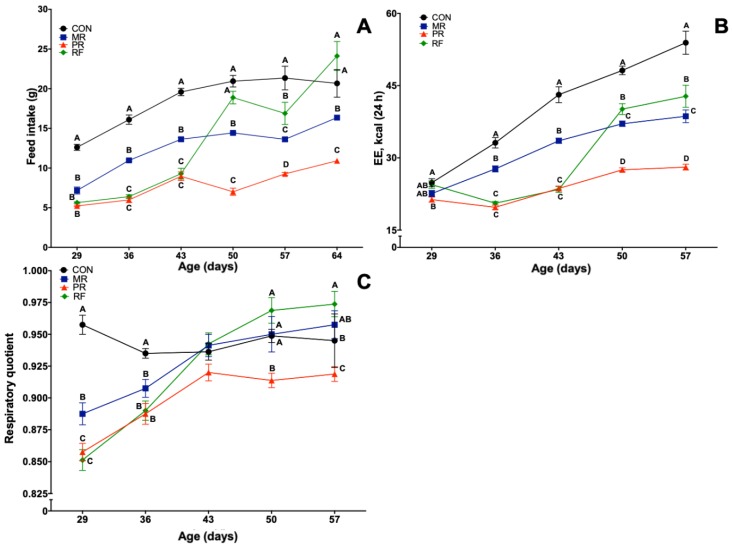
Mean (± SEM) effects of feed restriction and refeeding in rats on: feed intake (**A**); energy expenditure (**B**); and respiratory quotient (**C**) (n = 8/group). CON (Control), ad libitum feeding; MR (Mild Restriction), 75% of CON intake; PR (Profound Restriction), 50% of CON intake; and RF (Refeeding group), 50% of CON intake for 14 days, and then ad libitum. Treatment, time and treatment by time interaction were significant (*p* < 0.05) for all three end points. ^A–D^ Within a day of age, means without a common superscript differed (*p* < 0.05).

**Figure 2:**
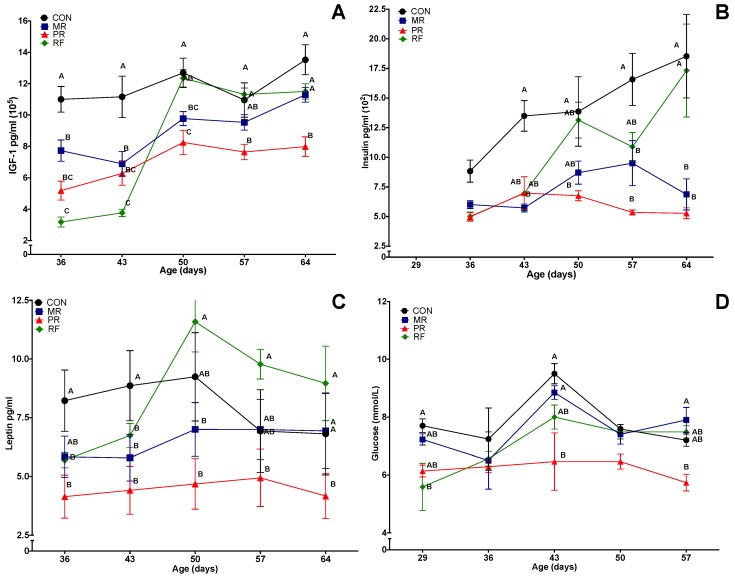
Mean (± SEM) effects of feed restriction and refeeding on plasma concentrations of metabolic hormones in rats (*n* = 8/group): (**A**) insulin-like growth factor-1 (IGF-1); (**B**) insulin; (**C**) leptin; and (**D**) glucose. CON (Control), ad libitum feeding; MR (Mild Restriction), 75% of CON intake; PR (Profound Restriction), 50% of CON intake; and RF (Refeeding group), 50% of CON intake for 14 days, and then ad libitum. Treatment, time and treatment by time interaction were significant (*p* < 0.001) for all three hormones and for glucose. ^A–C^ Within a day of age, groups without a common superscript differed (*p* < 0.05).

**Figure 3:**
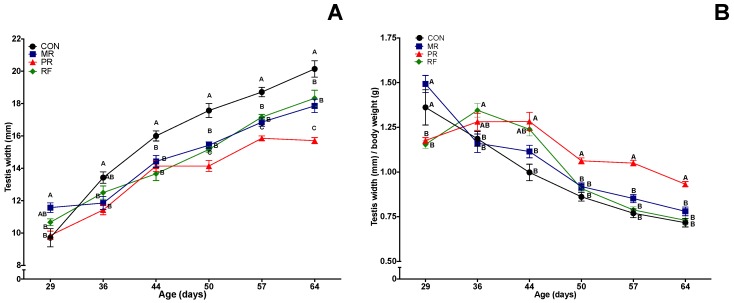
Mean (± SEM) effects of feed restriction and refeeding on testes width (absolute (**A**) and relative to body weight (**B**)) in rats (*n* = 8/group). CON (Control), ad libitum feeding; MR (Mild Restriction), 75% of CON intake; PR (Profound Restriction), 50% of CON intake; and RF (Refeeding group), 50% of CON intake for 14 days, and then ad libitum. Treatment, time and treatment by time interaction were significant (*p* < 0.001) for both end points. ^A–C^ Within a day of age, groups without a common superscript differed (*p* < 0.05).

Two-way analysis of variance for repeated measures (treatment, time and their interaction), followed by a Dunnet’s *t-*test, was used to analyze all data presented in Figure 1, Figure 2 and Figure 3. Energy expenditure was analyzed using (lean mass + 20% fat mass) as covariate [28]. For end points in Table 2 and Tables S1 and S2, one-way analysis of variance (treatment) for repeated measures, followed by a Dunnet’s *t-*test was used, except for evaluation of pubertal stage based on testosterone threshold (>1 ng/mL; Table 2) which was analyzed by Chi-square. Pearson’s correlation analysis was used to determine linear correlations between testicular width and testes weight, total sperm/testes, sperm/g testis, DSP and tubule diameter. All statistical analyses were performed with GraphPad Prism Version 6.0 (GraphPad Software Inc, La Jolla, CA, USA) and *p* < 0.05 was considered significant.

## 3. Results

### 3.1. Feed Intake, Energy Expenditure and Respiratory Quotient

For feed intake over five weeks, as a function of experimental design, there were effects of week, treatment and their interaction (*p* < 0.05; Figure 1A). As expected, rats in MR, PR and RF groups consumed less than CON, with rapid rebound of RF intake to CON levels on refeeding. For daily energy expenditure and RQ, there were effects of day, treatment and day × treatment interaction (*p* < 0.05) following a pattern similar to feed intake (Figure 1B,C). Total energy expenditures of MR, PR and RF groups were decreased during restriction, but, interestingly, on realimenation of RF, expenditure was restored to CON. Despite an initial reduction in RQ of MR, PR and RF groups, it gradually increased to CON levels in MR rats and was restored to CON levels on refeeding in RF rats.

### 3.2. Body Weight and Composition

There were effects of week, treatment and their interaction (*p* < 0.05) on body weight and composition. As expected, the PR group consistently had the lowest weight (*p* < 0.05) of all groups. Following refeeding, rats from the RF group rapidly gained body weight; although they had similar weight as the MR group after one week and were subsequently heavier than the MF group, they consistently remained lighter (*p* < 0.05) than CON rats. Profiles of body fat and lean followed the same pattern as body weight. At Day 64, body weight, fat and lean mass of PR were still lower than CON (*p* < 0.05) by ~39, 10 and 24 g, respectively.

### 3.3. Plasma Hormone and Glucose Concentrations

For all three metabolic hormones and glucose, there were effects of treatment, time and treatment × time (*p* < 0.001; Figure 2). In general, for serum concentrations of IGF-1, insulin and leptin, concentrations were highest for CON and lowest for PR on all days measured. In the RF group, as expected, hormone concentrations were similar to PR, but after refeeding (Day 43), they rebounded and were similar to CON rats.

For all three hormones, concentrations in the MR group were initially between CON and PR groups. As the study progressed, concentrations of IGF-I and leptin in the MR group approached those of the CON group, but insulin concentrations in the MR group were significantly lower than in the CON group during Days 50–64. For puberty based on testosterone >1 ng/mL, on Day 50, more CON and RF rats than PR rats had reached puberty (5/6, 4/5 and 1/6 respectively, *p* < 0.05) whereas the MR group (4/6) was not different from any other group (Table 2). However, plasma testosterone concentrations were >1 ng/mL in all rats by Day 64.

For weekly blood glucose concentrations (Figure 2D), there were effects of treatment and treatment × week (*p* < 0.05). For most days, blood glucose concentrations were lowest in the PR group, with no significant difference among the other three groups.

### 3.4. Testes Width and Weight

There were differences among groups in testis width at Day 29; thereafter, until the end of the study, testis width was consistently largest in the CON group and generally smallest in the PR group, with MR and RF groups between these two extremes and not significantly different from each other. When testes width was divided by body weight (Figure 3B), RF maintained similar values compared to the PR group (*p* < 0.05) during the first three weeks and reached similar values to the MR and CON groups at Day 50 (1 week of refeeding) onwards (*p* > 0.05). Thereafter, the three groups maintained similar values, whereas PR was different (*p* < 0.05) from all other groups through Day 64.

Testis width was significantly correlated with testes weight and various measures of sperm production (Table 2). Regarding paired testes weight, the PR group had smaller testes (*p* < 0.05) than all other groups (no significant difference among the latter; Table 3). However, for testes weight as a percentage of body weight, PR group had highest values, greater (*p* < 0.05) than CON and RF groups, whereas the MR group was not different from any other group (Table 2).

### 3.5. Sperm Motility, Morphology and Acrosome Integrity

There were no significant differences among groups for sperm motility (Table S2), morphology (Table S3) or acrosome integrity (Table S3).

### 3.6. mRNA Abundance of IGF-1

The mRNA abundance of IGF-1 and insulin-like growth factor 1 receptor (IGF-1R) in testis did not differ significantly among groups (Table S1).

## 4. Discussion

Although effects of nutrition on reproductive traits are well known in ruminants, swine and mice [9,29,30], e.g., reproductive and metabolic adaptations to calorie restriction during the peri-pubertal period remain largely unknown. To our knowledge, this was the first study to include a comprehensive assessment of metabolic and male reproductive parameters during the pubertal transition period. Herein, feed restriction delayed puberty and reproductive development in male rats, with degree of delay related to restriction severity. Notably, concurrent with delayed puberty, calorie restriction reduced energy expenditure and gains in lean and fat reserves, resulting in negative energy balance. However, based on plasma testosterone concentrations, puberty apparently occurred at varying body fat and lean reserves. Remarkably, in profoundly nutrient-restricted prepubertal rats (RF group), ad libitum feeding largely overcame negative effects of restrictive feeding and restored testis weight, number of sperm produced (per gram of testis and total), energy expenditure and plasma concentrations of IGF-1, leptin and insulin, although body composition end points had not completely recovered by the end of the study. Thus, there was general support for our hypothesis that nutrient restriction of pre-pubertal male rats delays reproductive development, induces negative energy balance and suppresses plasma concentrations of metabolic hormones, whereas short-term profound restriction and refeeding restores reproductive end points, energy expenditure and hormones to levels similar to those in control rats.

Refeeding rescued reproductive end points to levels similar to those in CON and MR groups. Commensurate with profound nutrient restriction in the PR group, there were substantial reductions in most reproductive end points; many had significant differences from all other groups. Notably, only 17% of PR rats were pubertal by 50 days of age in contrast to ~70–80% that attained puberty in other groups. Furthermore, PR rats had significantly lower testis weight, total sperm/testis, sperm/g of testis, DSP and smaller seminiferous tubule diameter. However, there was relatively greater suppression of testis width compared to body weight, resulting in a slower decline in this ratio in PR versus CON rats.

Few studies have used a pre-pubertal rat model to determine impact of nutritional restrictions on male fertility. In one study [4], male Sprague-Dawley rats were weaned at 21 days and immediately fed various diets (lowest intake group was ~30% ad libitum) for ~17.5 week. Profoundly underfed rats had reduced testis weight and diameter of seminiferous tubules, similar to our studies. In another study, from 3 to 7 weeks of age, male Wistar rats had a 20% reduction in feed intake, followed by ad libitum feeding for 4, 8 or 12 weeks [9]. In that study, after 4 weeks of refeeding, testicular weight was still significantly lower than the control group, although after 8 or 12 weeks of refeeding, all reproductive end points were similar to the control. Perhaps prolonged persistence of a reduction in testis weight in that study compared to the present study was due to differences in duration of restriction or rat strain. In another study, adult male rats were fed 15 g/day (moderate restriction) or 5 g/day (intense restriction) of regular chow for eight weeks; rats with moderate restriction had similar results as the control (ad libitum) but intense restriction reduced body and testes weight, fertility rate and litter size [31]. Outcomes of feed restriction using wild rodents were generally consistent with present results. Deer mice (*Peromyscus maniculatus*) with either 10% or 20% feed restriction had impaired mass of reproductive organs (testes, epididymis and seminal vesicle), spermatogenesis and sperm count (testicular and epididymal; [32]) and marsh rice rats limited to 80% or 60% of an ad libitum diet from 3 to 8 weeks of age had reduced paired testes weight [33].

Concurrent with robust changes in male reproductive indices, there were several important metabolic adaptations during feed restriction and after refeeding. As expected, reductions in gains of weight, fat and lean mass were proportional to degree of feed restriction. Similar to our findings, limiting intake to 90%, 80% or 70% of the control decreased weight gain in adult Sprague-Dawley rats [34]. In addition, feed restriction in rats reduced body fat content during and after puberty, testis weight and diameter of seminiferous tubules and serum and testicular testosterone concentrations, compared to control groups [4], similar to our study. Interestingly, that study [4] suggested that feed restriction would have less impact on spermatogenesis and age at puberty compared to testosterone concentrations, although in our study, the impact was equally severe for all of these end points. Despite 70–80% of CON, MR and RF rats being pubertal by 50 days, as defined by testosterone >1 ng/mL, rats in MR and RF groups had lower body weight, fat and lean mass than CON. Similarly, although all PR rats had testosterone >1 ng/mL at Day 64, their body fat and lean mass were also lower than CON. These novel findings from our rat model of calorie restriction supported the notion that puberty does not occur at a constant body composition in normal rats [4] and children [35,36].

Although it is well established that body composition determines EE (Energy Expenditure [28]) and that EE is reduced in feed-restricted animals [37], we are not aware of any studies that profiled EE during pubertal transition. This was apparently the first evidence that EE, after adjusting for body fat and lean mass, was dose-dependently decreased by calorie restriction during the prepubertal period. In the RF group, as expected, EE was low during feed restriction and then increased rapidly following refeeding, exceeding that of the MR group. This compensatory increase in total energy expenditure of PR likely contributed to delays in regaining weight and body tissue components with refeeding. Our findings of the gradual increase in EE in the CON group were, in general, consistent with increased total EE in pubertal adolescents in most studies [13], but in contrast to a longitudinal study that reported reduced resting EE during puberty in boys and girls [38]. There is an allometric relationship between testicular mass and body weight; smaller mammals direct a higher portion of EE to testicular function compared to larger mammals [15]. This relationship may account for feed restriction and subsequent refeeding having a substantial impact on testicular function in this study.

There is an important link between mechanisms regulating energy balance and reproductive success, since availability of adequate metabolites is fundamental to physiological functions of sexual behavior [39]. It is well documented that metabolic hormones including leptin, IGF-1 and insulin communicate level of body tissue reserves to the hypothalamic-pituitary-gonadal (HPG) axis [11,16,40], emphasizing importance of studying impacts of nutrition and refeeding on above-described hormones. In the present study, refeeding restored concentrations of IGF-I, insulin and leptin by Day 50 (7 days after being switched to an ad libitum diet) and sustained them to the end of the study. Furthermore, restricted rats had decreased serum concentrations of IGF-I, insulin and leptin, with concentrations significantly lower in PR than CON, whereas those in MR rats were intermediate.

Refeeding significantly increased plasma IGF-1 concentrations, maintaining values similar to controls until end of study. It is well established that IGF-I has great importance in male reproductive development. For example, in pre-pubertal Holstein bulls, IGF-1 was associated with an early (prepubertal) rise in serum LH concentrations and therefore reproductive onset [7]. Furthermore, similar to bulls, increased IGF-I seemed to promote reproductive development in the present study, as refeeding pre-pubertal rats overcame the previous restriction and restored testis weight and sperm production. Remarkably, there were no significant differences among groups in testicular expression of IGF-1 gene or its receptor. In contrast, there are indications regarding possible roles of IGF-1 and its receptor as stimulators of puberty and spermatogenesis in Holstein bull calves [5,7]. Interestingly, in our study, there were no differences among groups for mRNA abundance of insulin-like growth factor 1 (IGF-1) or insulin-like growth factor 1 receptor (IGF-1R) in testis. With the caveat that IGF-1 signaling intermediaries were not evaluated herein, perhaps IGF-1 is not as important in rats compared to bulls with regards to its effects on onset of puberty and supporting spermatogenesis.

Similar to IGF-1, leptin has also very important roles in supporting reproductive development, with a significantly higher concentration of this hormone on Day 64 for CON, MR and RF when compared to the PR group. In male mice with congenital deficiency of leptin, exogenous leptin increased FSH concentrations, increased testis weight and seminiferous tubule diameter and improved sperm production [41], highlighting the importance of this hormone in male reproduction.

Insulin followed the pattern described for IGF-1 and leptin, increasing on Day 50 and maintaining concentrations similar to CON rats (*p* > 0.05) until the end of the study (Day 64). Insulin supports spermatogenesis, not directly through testes, but apparently acting via the HPG axis and normalizing concentrations of LH and testosterone [42]. In the present study, CON and RF rats had significantly higher plasma concentrations of testosterone and insulin than the PR group (at Day 50), indicating a potential association between these two hormones.

Our study was apparently the first to use calipers to measure testicular width in rats. To our knowledge, there is a lack of non-invasive methods to assess testes in laboratory rodents. As testis weight and tubule diameter and sperm production were significantly correlated with testicular width, we inferred that this has potential as a reliable and non-invasive method of testicular assessment.

In conclusion, our study provided important and novel data on concurrent reproductive and metabolic adaptations in response to calorie restriction during the pubertal transition in male rats. Profound nutritional restriction of prepubertal male rats suppressed plasma concentrations of reproductive and metabolic hormones, had deleterious effects on growth and reproductive development and induced a concurrent state of negative energy balance, with reduced accretion of lean and fat reserves. However, refeeding restored testes size, sperm production, onset of puberty, hormonal profiles of IGF-1, leptin and insulin, and energy expenditure, although body weight and composition were not fully restored. Underlying mechanisms by which caloric restriction engages the gut-brain-reproductive axis to modulate reproductive development and energy balance during the pubertal period, remain to be investigated.

## Figures and Tables

**Table 1:** Primer sequences for reference genes used for quantitative real-time reverse transcription PCR in rat tissues.

Gene	Primer Sequence (5′→3′)	Location on Template (bp)	GenBank Accession No.	Annealing Temperature (°C)	qPCR Efficiency (%)
IGF1	Forward: TCTGCCTCTGTGACTTCTTGA	321–341	NM_001082478.1	61.4	98
	Reverse: AGCCTGTGGGCTTGTTGAAG	515–496			
IGF1R	Forward: TCGGCATCAAACTCCTCCTC	1903–1922	NM_052807	61.3	93.8
	Reverse: GATGGGTATTTTGTCTTTGGAGCA	2058–2035			
B-Actin	Forward: GGATCAGCAAGCAGGAGTACGA	1148–1169	NM_031144	60	113.3
	Reverse: AACGCAGCTCAGTAACAGTCCG	1229–1208			

**Table 2:** Pearson correlations (r) between testes width and other end points in rats (*n* = 32).

End Point	r	*p* Value
Testes weight	0.70	<0.0001
Total sperm/testes	0.78	<0.0001
Sperm/g testis	0.537	<0.01
Daily sperm production	0.537	<0.01
Tubule diameter	0.364	<0.07

**Table 3:** Mean (± SEM) effects of feed restriction and refeeding on age at puberty and testicular and sperm end points in rats (*n* = 8/group; birth = Day 0).

	CON	MR	PR	RF
Paired testis weight (g)	3.25 ± 0.11 ^a^	3.02 ± 0.12 ^a^	2.52 ± 0.04 ^b^	2.76 ± 0.09 ^a^
Testis (g) relative to body weight (100 g)	1.14 ± 0.04 ^a^	1.32 ± 0.07 ^ab^	1.5 ± 0.07 ^b^	1.12 ± 0.16 ^a^
Pubertal at Day 50 (testosterone >1 ng/mL)	5/6 ^a^	4/6 ^ab^	1/6 ^b^	4/5 ^a^
Total sperm/g testis (10^7^)	10.70 ± 0.20 ^a^	11.0 ± 0.30 ^a^	8.60 ± 0.20 ^b^	10.40 ± 0.30 ^a^
Total sperm/testes (10^7^)	34.80 ± 1.10 ^a^	33.30 ± 2.0 ^a^	22.10 ± 5.20 ^b^	30.10 ± 1.0 ^a^
Daily sperm production (10^6^/g testis)	17.60 ± 0.40 ^a^	18.0 ± 0.60 ^a^	14.10 ± 0.30 ^b^	17.0 ± 0.50 ^a^
Seminiferous tubule diameter (µm)	436.80 ± 1.60	432.10 ± 1.76 ^a^	409.50 ± 1.52 ^b^	433.10 ± 1.98 ^a^

CON (Control), ad libitum feeding; MR (Mild Restriction), 75% of CON intake; PR (Profound Restriction), 50% of CON intake; and RF (Refeeding group), 50% of CON intake for 14 days, and then ad libitum. ^a, b^ Within a row, means without a common superscript differed (*p* < 0.05).

## Supplementary Materials

The following are available online at https://www.mdpi.com/2072-6643/11/9/1993/s1, Table S1. Fold change in expression of selected genes, effects of feed restriction and refeeding on mRNA abundance of key regulatory molecules in testes of young rats (*n* = 8/group). CON (Control), ad libitum feeding; MR (Mild Restriction), 75% of CON intake; PR (Profound Restriction), 50% of CON intake; and RF (Refeeding group), 50% of CON intake for 14 days, and then ad libitum. Table S2. Mean (± SEM) epididymal sperm motility parameters at Day 64 (birth = Day 0) in rats fed various diets from Days 29 to 64 (8 rats per group). Table S3. Mean (± SEM) epididymal sperm morphology and acrosome integrity at Day 64 (birth = Day 0) in rats fed various diets during Days 29–64 (8 rats/group).
