# Supplementary material for: Calorie Restriction Modulates Reproductive Development and Energy Balance in Pre-Pubertal Male Rats
Source: Nutrients. 2019 Aug 23;11(9):1993. doi: 10.3390/nu11091993 (PMC6770304; doi:10.3390/nu11091993)
Supplement: Supplementary file 1 [file nutrients-11-01993-s001.pdf]

*Supplementary*

**Table S1.** Fold change in expression of selected genes, effects of feed restriction and refeeding on mRNA abundance of key regulatory molecules in testes of young rats ($n$ = 8/group). CON (Control), ad libitum feeding; MR (Mild Restriction), 75% of CON intake; PR (Profound Restriction), 50% of CON intake; and RF (Refeeding group), 50% of CON intake for 14 days, and then ad libitum.

| Groups | CON | MR | PR | RF |
|---|---|---|---|---|
| IGF-1 | - | 0.76 | 1.53 | 0.61 |
| IGF-1R | - | 1.11 | 0.75 | 0.35 |

**Table S2.** Mean (± SEM) epididymal sperm motility parameters at Day 64 (birth = Day 0) in rats fed various diets from Days 29 to 64 (8 rats per group).

| | CON | MR | PR | RF |
|---|---|---|---|---|
| **Motile (%)** | 43.08 ± 3.33 | 46.11 ± 3.66 | 40.30 ± 3.46 | 43.08 ± 3.33 |
| **Progressive (%)** | 26.20 ± 3.21 | 29.22 ± 3.3 | 22.88 ± 2.8 | 22.1 ± 2.58 |
| **Immobile** | 55.74 ± 3.58 | 53.82 ± 3.87 | 60.97 ± 2.89 | 58.32 ± 2.66 |
| **Hyperactive** | 7.99 ± 1.37 | 9.77 ± 2.59 | 12.92 ± 4.68 | 8.12 ± 1.53 |
| **Linear** | 11.98 ± 1.72 | 14.01 ± 1.76 | 11.82 ± 1.96 | 13.21 ± 2.23 |
| **Non-linear** | 10.42 ± 2.71 | 9.62 ± 2.44 | 7.83 ± 1.43 | 9.4 ± 1.34 |
| **Curvilinear** | 6.28 ± 1.06 | 5.31 ± 0.64 | 4.29 ± 0.46 | 6.51 ± 1.69 |

CON (Control), ad libitum feeding; MR (Mild Restriction), 75% of CON intake; PR (Profound Restriction), 50% of CON intake; and RF (Refeeding group), 50% of CON intake for 14 days, and then ad libitum.

**Table S3.** Mean (± SEM) epididymal sperm morphology and acrosome integrity at Day 64 (birth = Day 0) in rats fed various diets during Days 29–64 (8 rats/group).

| End Point | CON | MR | PR | RF |
|---|---|---|---|---|
| **Normal** | 95.31 ± 1.10 | 96.43 ± 1.53 | 93.78 ± 3.7 | 94.08 ± 1.6 |
| **Bent tail** | 1 | 1.16 ± 0.31 | 1.5 ± 0.71 | 1 ± 0.58 |
| **Distal droplet** | 1.21 ± 0.81 | 0.83 ± 0.17 | 2.33 ± 1.83 | 0.63 ± 0.2 |
| **Coiled tail** | 0 | 0.6 ± 0.37 | 0.83 ± 0.44 | 0.5 |
| **Detached head** | 2.25 ± 0.92 | 1.21 ± 0.19 | 2.28 ± 1.91 | 3.75 ± 1.48 |
| **Bent neck** | 2 ± 1.48 | 1.13 ± 1.19 | 0.83 ± 0.22 | 1.1 ± 0.18 |
| **Flat head** | 0.5 | 1 | 1 | 0.5 |
| **Abnormal tail** | 1.44 ± 0.99 | 1.64 ± 0.97 | 3.43 ± 2.16 | 1.17 ± 0.8 |
| **Abnormal head** | 3.31 ± 1.24 | 2.0 ± 0.69 | 2.79 ± 2.13 | 4.75 ± 1.59 |
| **Acrosome integrity** | 98.87 ± 1.12 | 99.0 ± 0.38 | 99.07 ± 0.43 | 99.08 ± 0.13 |

CON (Control), ad libitum feeding; MR (Mild Restriction), 75% of CON intake; PR (Profound Restriction), 50% of CON intake; and RF (Refeeding group), 50% of CON intake for 14 days, and then ad libitum.
